# Validation of a Self-Regulated Foreign Language Learning Strategy Questionnaire Through Multidimensional Modelling

**DOI:** 10.3389/fpsyg.2018.01388

**Published:** 2018-08-06

**Authors:** Anita Habók, Andrea Magyar

**Affiliations:** ^1^Institute of Education, University of Szeged, Szeged, Hungary; ^2^Center for Research on Learning and Instruction, University of Szeged, Szeged, Hungary

**Keywords:** self-regulated learning, foreign language learning, language learning strategy, validation, multidimensional modelling

## Abstract

The main objective of the present research is to validate a Self-Regulated Foreign Language Learning Strategy Questionnaire based on previous research, conceptualised in a strategy inventory for language learning and self-regulated language learning. A total of 2223 lower secondary school children participated in the study. After the questionnaire development process, children completed the questionnaire online. Confirmatory factor analyses (CFA) were conducted through structural equation modelling (SEM) to assess our hypothesised six-factor structure model. The results of the CFA validated a five-factor correlated model with metacognitive, cognitive, meta-affective, meta-sociocultural-interactive and sociocultural-interactive factors, while the affective factor was not included. Internal and composite reliability confirmed the consistency of our factors, and convergent validity provided evidence for significant relationships between them. Our results draw attention to the complexity of language learning strategy use, which spans cognitive, affective and sociocultural factors as well as their ‘meta’ approaches. A more concrete distinction demands further investigation and a more accurate design of the questionnaire in the affective field.

## Introduction

During the past 30 years, the concept of language learning strategies (LLS) has become fundamental in foreign language learning, and a vast number of studies have dealt with establishing various definitions, interpretations, categorisations and measurement tools (O’Malley and Chamot, 1990; Oxford, 1990; Cohen, 1996, 2007; Chamot, 2004; Nagy and Habók, 2018). One of the best-known instruments is the Strategy Inventory for Language Learning (SILL), developed by Oxford (1990). However, the psychometric properties of the assessment instruments have recently been questioned (Dörnyei, 2005; Tseng et al., 2006); therefore, the original concept was reconsidered and the classification of the strategies was restructured based on self-regulated learning (SRL) theory (Oxford, 2011). Oxford (2011, 2017) aimed to bridge the gaps between language learning strategies theory and self-regulated learning with her Strategic Self-Regulation (S2R) Model of language learning and thus established new perspectives in strategy research.

Our aim was to reconsider SILL in the light of her newly improved model, to develop an alternate version based on the multidimensional structure of the S2R model and to validate this measurement tool empirically among lower secondary English as foreign language (EFL) students. In the first part of the paper, we outline the most important steps that led to the introduction of the new paradigm in language learning strategies research, then introduce the phases of the development of Oxford’s restructured questionnaire and finally present the validation process of the assessment tool.

## Theoretical Background

### The Conceptualisation of LLS and SRL

Since Rubin (1975) started to determine the characteristics of good and successful language learners, the strategic view of language learning has gained increasing attention. Scholars began to identify, define and classify strategies, and a large variety of trends have developed. Since 1975, Oxford (2017) has listed 33 different definitions for the terms ‘language learning strategies,’ ‘learner strategies,’ ‘self-regulated learning strategies,’ ‘strategies,’ and ‘strategic.’ In this study we follow Oxford’s widely accepted definition (1990): LLS are ‘specific actions taken by the learner to make learning easier, faster, more enjoyable, more self-directed, and more transferrable to new situations’ (p. 8).

Like the vast number of definitions, a large number of classifications have also evolved. For several years, Oxford’s (1990) six-category strategy taxonomy of direct (memory, cognitive, and compensation) and indirect strategies (metacognitive, affective, and social) was widely accepted and used; in recent years, however, some researchers have argued that the terms used for language learning strategies are too general and diverse, and not clearly defined (e.g., Dörnyei, 2005; Tseng et al., 2006). With regard to Oxford’s taxonomy, Dörnyei (2005) proposed that compensatory strategies are rather linked to language use than to language learning. The separation of cognitive and memory strategies was also criticised because memory strategies rather ‘constitute a subclass of cognitive strategies’ (Dörnyei, 2005, p. 168). In addition, Dörnyei (2005) proposed eliminating the term ‘strategies’ and replacing it with ‘self-regulation.’

The notion of self-regulation originates from the field of educational psychology and enjoys a long tradition. Since the 1980s, the concept of self-regulation has been studied through diverse theoretical perspectives (de la Fuente-Arias, 2017). Pintrich (1995) was the first scholar to define ‘self-regulated learning’ as an active and constructive process. During this process students create learning aims and manage, organise and supervise their actions accordingly to achieve these goals. A large number of models have been constructed on the basis of this definition. Panadero (2017) collected, compared and reviewed the six most widely acknowledged models. Most of the SRL models incorporate cognitive, metacognitive, behavioural, motivational and affective dimensions of learning, and cover a vast number of variables, e.g., self-efficacy, self-efficiency, metacognitive and cognitive strategies, motivational and emotional factors, and learner’s beliefs.

As self-regulated strategies relate to language learning strategies, Dörnyei (2005) assumed that involving self-regulation in the language learning process would lead to a broader understanding of the notion than recent definitions of LLS. With this change, emphasis has been placed on the process rather than the product. In addition, he also stated that self-regulation represents the basis for a more dynamic model than language learning strategy concepts.

### Strategic Self-Regulation Model of Language Learning (S2R Model)

Recently, the lack of theoretical consensus has led Oxford to reconsider her original concept and incorporate self-regulation theory into her model. In her Strategic Self-Regulation (S2R) Model, self-regulated learning strategies have been specified as deliberate, goal-directed attempts to control and manage the foreign language learning process. She regarded these strategies as teachable actions that language learners choose from among several choices and employ to support their L2 learning purposes (e.g., constructing, adopting, storing or using information for various purposes and/or developing their L2 proficiency and self-efficacy in the broader sense) (Oxford, 2011).

Oxford (2011, 2017) incorporated three key dimensions of language learning into her model: the cognitive, affective and sociocultural-interactive fields. Cognitive strategies are defined as strategies that ‘help the learner construct, transform, and apply L2 knowledge’ (p. 14) (e.g., activating knowledge). Affective strategies are those that ‘help the learner create positive emotions and attitudes and stay motivated’ (p. 14) (e.g., generating and maintaining motivation). Sociocultural-interactive (SI) strategies ‘help the learner with communication, sociocultural contexts, and identity’ (p. 14) (e.g., interacting to learn and communicate). She places six strategies in the cognitive field, two in the affective category and three in SI. These strategies are guided by metastrategies (metacognitive, meta-affective, and meta-SI), which serve as conductors in an orchestra. They control and manage the language learning process, as well as support and regulate the learner’s needs in diverse contexts and situations. She distinguishes eight metastrategies: paying attention, planning, obtaining and using resources, organising, implementing plans, orchestrating strategy use, monitoring and evaluating. She includes a total of 19 strategies in her self-regulated L2 learning model (Oxford, 2011).

By introducing this *metastrategic regulation*, she expanded Flavell’s (1979) cognitive monitoring model and incorporated self-regulation into her theory (Oxford, 2011). Expanding the domain of control of strategies in the affective and SI fields is the most important outcome of her S2R theory.

### Assessment Tools for Measuring LLS and SRL

During the past 20 years, a vast number of assessment tools have been developed to assess both LLS (Oxford, 1990; Tseng et al., 2006; Zhang and Seepho, 2013; Teng and Zhang, 2016) and self-regulation (Zimmerman and Kitsantas, 2002; Bruning et al., 2013; Artuch-Garde et al., 2017; De la Fuente et al., 2017). For several decades, the most widely accepted and most widespread measurement tool was the Strategy Inventory for Language Learning (SILL) developed by Oxford (1990). There are two versions: a 50-item self-report inventory for learners of English and an 80-item version for speakers of English learning other languages. The 50-item questionnaire is divided into six strategy fields: (1) memory (9 items), (2) cognitive (14 items), (3) compensation (6 items), (4) metacognitive (9 items), (5) affective (6 items) and (6) social strategies (6 items). The learners rate items on a five-point scale ranging from 1 (‘Never or almost never true of me’) to 5 (‘Always or almost always true of me’). The reported internal consistency reliabilities of the questionnaires ranged between 0.91 and 0.94 (Cronbach’s alpha) (Oxford and Burry-Stock, 1995). Recently, in line with criticism of the LLS, the psychometric characteristics of the assessment instruments were also seriously criticised by some researchers (Dörnyei, 2005; Woodrow, 2005; Rose, 2011; Amerstorfer, 2018). Woodrow (2005) argued that the standard Likert-type scales that are employed in SILL are inaccurate and unreliable because the wide range of contextual differences, such as cultural or educational background, can greatly influence the results. Instead, the quantitative assessment he proposed shifted toward in-depth qualitative methods. Further, Dörnyei (2005) argued that the rating scales used in SILL are based on frequency of use, and he questioned their psychometric indicators. They also criticised the lack of a direct relationship between the frequencies of strategy use and learning success.

In line with Oxford’s reconsidered S2R theory, several measurement tools have also been developed on the basis of SILL (Rose et al., 2017). Wang et al. (2013) constructed the Questionnaire of English Self-Regulated Learning Strategies (QESRLS), in which they incorporated the S2R model. Their 67-item questionnaire comprised 12 categories: self-evaluation (4 items), organising and transforming (18 items), rehearsing and memorising (5 items), seeking social assistance (3 items), persistence (3 items), seeking opportunities (8 items), taking records (2 items), self-consequence (2 items), goal-setting (3 items), reviewing records (2 items), use of native language (6 items), and interpretive guessing (5 items). The internal consistency (Cronbach’s alpha) of the measurement tool was high (0.96), and test–retest reliability was also high (0.88). The data for the validation study were collected among university students.

Salehi and Jafari (2015) also developed a Self-Regulated Learning Questionnaire focusing on language learners’ SRL capacity and behaviour. The 41-item questionnaire contains 13 sub-scales: intrinsic motivation, self-efficacy, attitude, organisation, memory strategies, self-monitoring, planning and goal-setting, effort regulation, regulation of environment, help seeking, locus of control orientation, concentration and sustained attention. Except for memory strategies, the Cronbach reliabilities of the questionnaire ranged from 0.70 to 0.94. The ages in the sample were between 14 and 47.

De la Fuente et al. (2015) investigated how personal self-regulation and regulatory teaching processes are related to learning approaches and how we can predict motivational-affective variables, academic achievement and satisfaction. Their findings point out the significance of interactive relationships in the teaching and learning processes, and the significance of personal self-regulation.

All these scales consisted of several subscales and items. They were therefore complicated to use in the classroom environment, and they were mostly validated with university students or adults at an intermediate level of English or above. The scales did not consider the strategy use of students at the beginner or elementary level. Another drawback was that they did not take the different cultural backgrounds of the learners into consideration.

Some studies have aimed to reconsider SILL based on Oxford’s S2R taxonomy and adjust it to their specific national, cultural, social, or educational contexts. Seker (2015) developed a 30-item self-regulated language learning questionnaire for the Turkish context, which was administered among Turkish university students at Level A2. Exploratory Factor Analysis (EFA) resulted in five factors that largely corresponded to the five sub-constructs in his concept.

Chen and Lin’s (2018) research re-validated Carey et al.’s. (2004) Short Self-Regulation Questionnaire (SSRQ) based on a national sample of Taiwanese college students. The Taiwanese Short Self-regulation Questionnaire (TSSRQ) was successfully employed to various contexts, and it was also used to deal with different issues beyond learning.

Köksal and Dündar (2017) also developed a scale for the Turkish context. It consisted of 35 items embedded in the six factors in Oxford’s S2R Model (2011). The reliability of the questionnaire showed an acceptable level with a Cronbach’s alpha statistic of 0.85. Both EFA and Confirmatory Factor Analysis (CFA) confirmed the construct validity of the scale.

Božinović and Sindik (2017) constructed a modified version of SILL specifically for the Croatian context. The questionnaire contained 55 items. It eliminated compensation strategies, merged social and affective strategies into social-affective strategies, and inserted a special section for grammar learning strategies.

These results have provided support for the suitability of using the construct of self-regulation in language learning. However, in Hungary specifically, there is a dearth of research into learning strategies, particularly in the elementary school learning context. Recent studies show a desperate need in this area, especially for children with low and average language proficiency (Magyar and Habók, 2016; Habók and Magyar, 2018).

## Research Question

The present research aims to validate the Self-Regulated Foreign Language Learning Strategy Questionnaire (SRFLLSQ). Our main research goal was to explore whether the structural model represents the dimensions of S2L in the sample for Years 5 and 6. We assumed that the strategies would be classified into six factors (metacognitive, cognitive, meta-affective, affective, meta-sociocultural-interactive, and sociocultural-interactive) covering the self-regulated learning strategy fields.

## Procedure

### Participants

The research involved a total of 2223 children from Hungarian lower secondary schools (**Table 1**). The students have been learning English for 2 or 3 years. There were no exclusion criteria in the sample. All the children who completed the questionnaire were included in the survey, since all their data was valuable.

**Table 1:** Characteristics of the sample.

Year	Gender			Total
	Boys	Girls	Absent	
5 (11-year-olds)	582	598	36	1216
6 (12-year-olds)	458	527	22	1007
Total	1040	1125	58	2223

### Instrument

The SRFLLSQ questionnaire was developed through a three-phase process. First, we analysed the related theoretical background and reviewed existing questionnaires in the fields of SRL and LLS. Second, we concluded that parts of SILL may be suitable for our measurement tool. After selecting the related items from SILL, one researcher compiled items that may be relevant for our lower secondary school sample. Next, another researcher reviewed the list of items and added items from the relevant SRL literature. Finally, we discussed all the items as a whole, fitted these to the construct involved in the research and completed the questionnaire. A 34-item questionnaire was then developed (**Appendix**). The complex questionnaire was assigned to the strategy fields from Oxfords’ Strategic SRL model: metacognitive (8 items), cognitive (6 items), meta-affective (7 items), affective (2 items), meta-sociocultural-interactive (8 items) and sociocultural-interactive (3 items). A five-point Likert scale was used for the children’s responses. The scale ranged from 1 (‘Never or almost never true of me’) to 5 (‘Always or almost always true of me’).

### Design

The study was conducted in accordance with the recommendations of the University of Szeged. The IRB at the Doctoral School (University of Szeged) specifically approved this research. The required written consent forms are held by the schools. Partner schools received a call to register for the testing procedure if they wanted to participate in the study. The schools that sent us feedback on participation were contacted and received instructions as well as the questionnaire link for data collection. Since the data for the schools were handled through central administration, the researchers have no information about which school was involved in the measurement at the national level. The responses are treated confidentially and are not disclosed to third parties. They are identified by a separate administrator at the university.

The questionnaire was administered through the Online Diagnostic Assessment System (eDia), which was developed and operated by the University of Szeged Centre for Research on Learning and Instruction. This platform is an internet-based interface in that both teachers and children are able to follow the online data collection process and obtain the results immediately. Only basic ICT skills are necessary to use the system. The platform not only allows progress to be recorded, but also collects background data on the process, such as response time. The platform identifies each user’s code, and only one response is allowed.

Students were provided one school lesson to complete the questionnaire, and they were able to work independently on the online system. They clicked on the radio button to indicate their choices on the Likert scale. The teacher only helped to eliminate any technical problems that arose. On average, the children needed 15 min to complete the questionnaire. Data was recorded twice: after piloting, we also collected data on a large-scale sample.

### Data Analysis

Data analysis occurred in two steps. First, confirmatory factor analyses (CFA) were conducted through structural equation modelling (SEM) to evaluate our hypothesised model based on the strategic self-regulation model. For the analysis the IBM SPSS AMOS 23.0 was used. The following goodness of fit indices were used to evaluate model fit: Chi-square test, comparative fit index (CFI), Tucker–Lewis Index (TLI), root mean square error of approximation (RMSEA) and KMO index (Kline, 2015). Chi-square statistics aided us in selecting the appropriate structural model among the hypothesised nested models. The difference in chi-square as a ratio of the difference in df was examined with the significance of the *p*-value. As Kline (2015) stated, the chi-square test statistic is sensitive to sample size and significant chi-square values are regularly found when large samples are involved; we therefore also regarded CFI values, as they are not sensitive to sample size. They range from 0 to 1, with larger values indicating a better fit. A regularly larger value than 0.90 indicates an acceptable model fit. The RMSEA is also a critical value during the analysis because it calculates the model fit while also regarding the complexity of the model structure. A value of 0.08 or less usually indicates a good model fit. A KMO test indicates the appropriateness of data for factor analysis. Factor loadings are regarded to be high when they are higher than 0.60. In our study, we considered a factor loading of 0.80 or higher as significant.

Second, IBM SPSS Statistics 23.0 and the R package were used for classical test analysis to ascertain reliabilities, mean, standard deviation and correlation. The internal consistency reliabilities (Crbα; Cronbach’s alpha) and the composite reliabilities (CR; McDonald’s coefficient omega; Raykov, 1997) were estimated to evaluate reliability. Values above 0.70 indicate good results for empirical research (Hair et al., 2010). The construct validity of the measurement model was accessed through convergent and discriminant validities. Convergent validity evaluates the degree to which items in a theoretical model relate to each other. It is confirmed when all factors in the same construct are higher than 0.70. Additionally, the CR for each construct should be larger than 0.70, and average variance extracted (AVE) should be higher than 0.50. However, lower values are also acceptable when the CR values are higher than 0.60 (Fornell and Larcker, 1981). Discriminant validity ascertains whether items belonging to a construct can be distinguished from those of another construct. The heterotrait-monotrait ratio of correlations (HTMT) was used to assess discriminant validity. This is the average of the heterotrait-heteromethod correlations relative to the average of the monotrait-heteromethod correlations (Henseler et al., 2015, p. 121). We employed HTMT as a criterion, which involved comparing it to a predefined threshold. According to Kline (2015), a threshold value of 0.85 is an acceptable eligibility criterion for discriminant validity. If the value of the HTMT is lower than this threshold, we can confirm discriminant validity.

## Results

### Confirmatory Factor Analysis (CFA)

**FIGURE 1:**
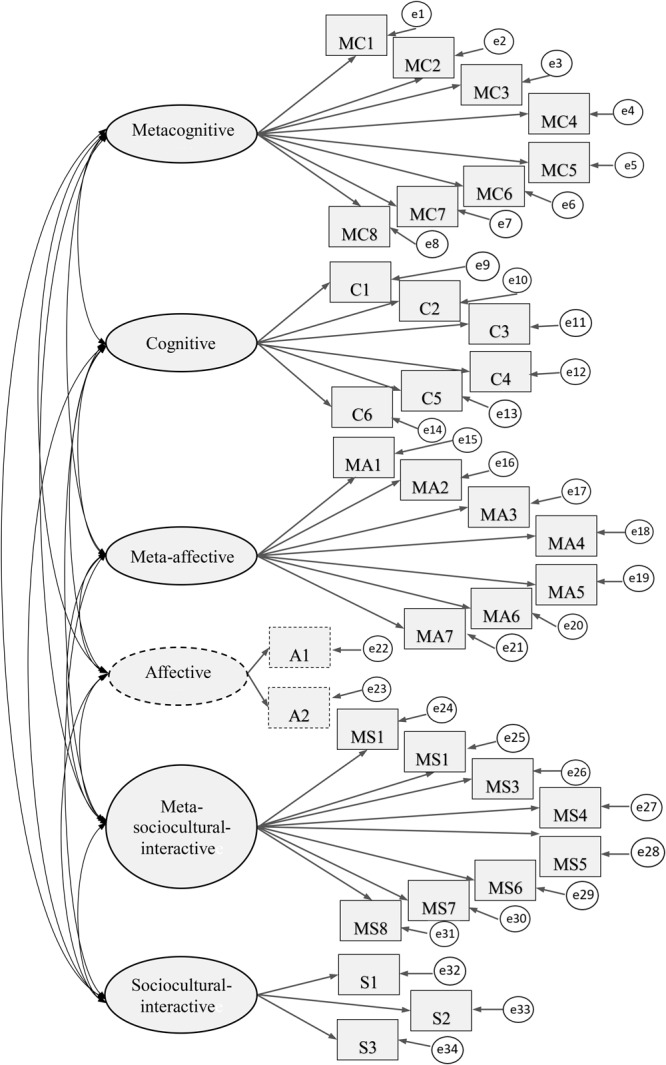
The six-factor structural model of the SRFLLSQ, displaying latent and observed variables and measurement errors (*N* = 2223).

Based on Oxford’s (2011) theoretical design, we first analysed the six-factor model (see **Figure 1**).

In the structured model, one-headed arrows show hypothesised one-way directions; two-headed arrows represent the correlation between two variables, in this case, between strategy fields. Ovals represent latent variables (i.e., a questionnaire factor), rectangles show observed variables (i.e., a questionnaire item), and small circles are used to indicate measurement errors specific to each of the observed indicators.

**FIGURE 2:**
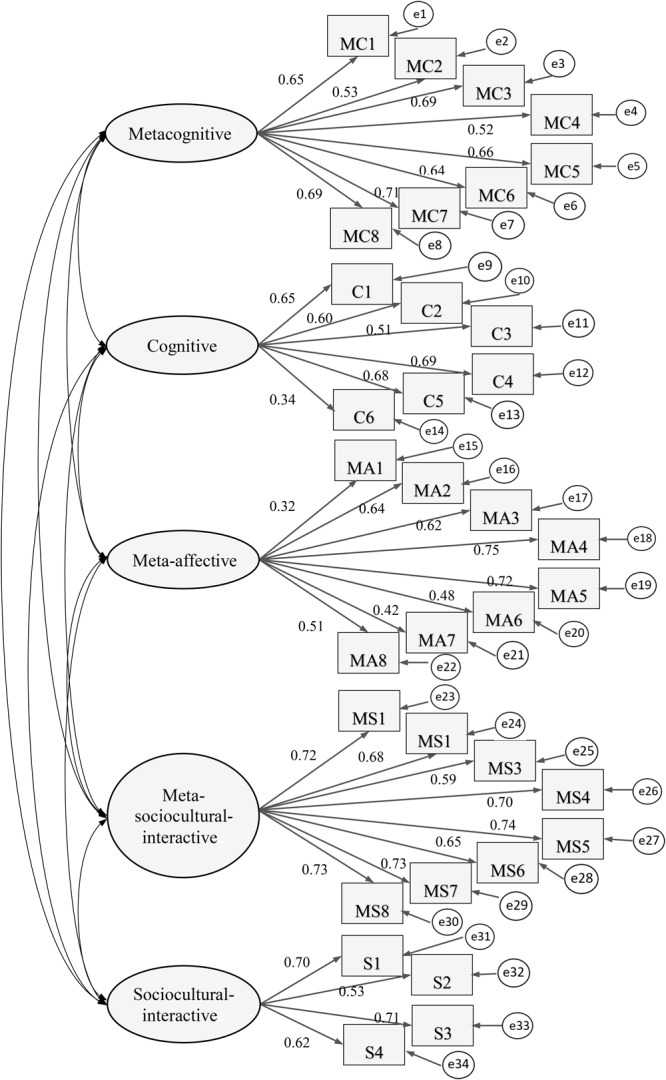
The five-factor structural model of the SRFLLSQ, displaying latent and observed variables and measurement errors (*N* = 2223).

The results for the years were handled together since there were only slight differences in their strategy use. We assumed that we would be able to describe the questionnaire fields. The results from a confirmatory factor analysis (CFA) indicated an unacceptable model fit, and our model did not fit our data because the affective dimension indicated unfitted estimates. Hence, we eliminated the affective dimension and incorporated the two items into the meta-affective and sociocultural-interactive fields based on their content. This time, our CFA therefore showed an acceptable model fit (Chi-square = 3137; df = 517; *p* = 0.000; CFI = 0.912; TLI = 0.899; RMESA = 0.048). **Figure 2** represents the standardised result for the five-factor model.

All the fields also showed appropriate fit indices separately. **Table 2** summarises the fit indices for the questionnaire fields.

**Table 2:** Goodness of fit indices for questionnaire fields.

Strategy	Chi-square	df	*p*<	CFI	TLI	RMSEA
Metacognitive	165	20	0.001	0.972	0.949	0.057
Cognitive	27	9	0.01	0.993	0.983	0.030
Meta-affective	240	20	0.001	0.941	0.894	0.070
Meta-sociocultural-interactive	175	20	0.001	0.997	0.958	0.059
Sociocultural-interactive	23	2	0.001	0.987	0.937	0.069

### Reliability

Internal consistency reliabilities were computed for each of the fields (**Table 3**). Both Cronbach’s alpha and omega coefficients for each were acceptable for all five factors. Their values ranged between 0.74 and 0.88 on the five subscales, suggesting satisfactory reliabilities. The meta-sociocultural-interactive strategy field indicated the highest reliability (Crbα = 0.88; ω = 0.88), while the metacognitive field was also high (Crbα = 0.84; ω = 0.84). Cronbach’s alpha and omega coefficients for the meta-affective (Crbα = 0.77; ω = 0.79) and sociocultural-interactive (Crbα = 0.74; ω = 0.74) fields fell slightly below the level of acceptability. The cognitive field also showed acceptable coefficients (Crbα = 0.75; ω = 0.76). Our KMO index was very high at 0.972. This shows that our data was adequate for a factor analysis.

**Table 3:** Internal consistency reliability (CRB) and composite reliability (CR).

Strategy	CRB	CR
Metacognitive	0.84	0.84
Cognitive	0.75	0.76
Meta-affective	0.77	0.79
Meta-sociocultural-interactive	0.88	0.88
Sociocultural-interactive	0.74	0.74

### Validity

Average variance extracted values were calculated to confirm the convergent validity of the scales. They showed slightly lower values for all factors ranging from 0.33 to 0.48. While the CR values were higher than 0.60 (Fornell and Larcker, 1981), these relatively low values are also acceptable. Additionally, our results showed a strong significant correlation between the five factors. The inter-correlation coefficients (*r*) ranged from 0.63 to 0.75. The composite reliability is higher than 0.7 for all constructs in the measurement model (**Table 3**), so convergent validity is confirmed (**Table 4**).

**Table 4:** Average variance extracted (AVE) and inter-correlations for the 5-factor correlated model.

Strategy	AVE	MC	C	MA	MS	S
Metacognitive (MC)	0.41		0.71	0.70	0.70	0.66
Cognitive (C)	0.35			0.68	0.64	0.63
Meta-affective (MA)	0.33				0.72	0.68
Meta-sociocultural-interactive (MS)	0.48					0.75
Sociocultural-interactive (S)	0.42					

*MC, metacognitive; C, cognitive; MC, meta-affective; MS, meta-sociocultural-interactive; S, sociocultural-interactive. All correlations are significant at p < 0.001*.

Discriminant validity was assessed using the HTMT ratio (Henseler et al., 2015). **Table 5** indicates the results. The values ranged from 0.63 to 0.75. All values are less than 0.85, so discriminant validity is confirmed.

**Table 5:** HTMT ratio of the correlations for the factors.

Strategy	MC	*C*	MA	MS	*S*
Metacognitive (MC)		0.71	0.70	0.70	0.66
Cognitive (C)			0.68	0.64	0.63
Meta-affective (MA)				0.72	0.69
Meta-sociocultural-interactive (MS)					0.75
Sociocultural-interactive (S)					

### Means and SDs for the 2 Years

Descriptive statistical analysis indicated that the mean scores for the 34 items ranged from 3.45 to 3.68 in Year 5 and from 3.28 to 3.63 in Year 6 (**Table 6**). In four cases, we found no significant differences between the years. Only meta-affective strategy use was significantly higher in Year 5. The highest strategy use was found in the meta-sociocultural-interactive field. The lowest was observed in cognitive and meta-affective strategy use in Year 5 and in the meta-affective field in Year 6.

**Table 6:** The strategy use results for the two subsamples.

	Year 5		Year 6		*t*	*p*<
Strategy	Mean	*SD*	Mean	*SD*		
Metacognitive	3.58	0.80	3.51	0.75		n.s.
Cognitive	3.45	0.81	3.39	0.74		n.s.
Meta-affective	3.45	0.82	3.28	0.75	5.036	0.001
Meta-sociocultural-interactive	3.68	0.92	3.63	0.86		n.s.
Sociocultural-interactive	3.64	0.98	3.59	0.91		n.s.

## Discussion

The main objective of the research was to validate a self-reported Self-Regulated Foreign Language Learning Strategy Questionnaire. The item framing started with a review of Oxfords’ SILL, followed by item construction. The questionnaire was completed according to the Strategic Self-Regulation model. The results of the CFA provided substantial evidence for the factorial structure of the questionnaire. We identified five distinct factors: metacognitive, cognitive, meta-affective, meta-sociocultural-interactive and sociocultural-interactive. The affective factors did not show acceptable fit indices; we therefore integrated the two items from this field into the meta-affective and sociocultural-interactive fields. Our model fitted to the data with these changes, and the fields had acceptable internal reliabilities and fit indices. The various psychometric analyses provided evidence that the construct for the scale was suitable and meaningful.

The final factor structure of the 34-item questionnaire was the following (**Appendix**): cognitive (6 items), sociocultural-interactive (4 items), metacognitive (8 items), meta-affective (8 items), and meta-sociocultural-interactive (8 items). According to Oxford’s definition (2011), cognitive strategies enable learners to ‘construct, transform, and apply L2 knowledge’ (p. 14) (e.g., *I find the meaning of an English word by dividing it into parts that I understand*). Sociocultural-interactive strategies are tied to ‘communication, sociocultural contexts, and identity’ (Oxford, 2011, p. 14) (e.g., *When I speak with highly proficient speakers of English, I think it is important to get acquainted with their culture*). Metastrategies enable learners to ‘control and manage the use of strategies in the three other dimensions: cognitive, affective, and sociocultural-interactive’ (Oxford, 2011, p. 15) (e.g., *I look for similarities and differences between my own culture and the cultures of English native speakers and/or other cultures through English*).

The factor structure somewhat resembled Köksal and Dündar’s (2017) 35-item scale embedded in six factors, Seker’s (2015) 30-item, five-factor questionnaire, and Božinović and Sindik’s (2017) scale of four plus one factors. As with our results, the affective dimension was also eliminated in Božinović and Sindik’s (2017) study. They merged it with the social subscale, which is also in line with our findings.

As regards the results of the validity assessments, items tied to a particular theoretical construct relate well to each other, and items that form a construct can be distinguished adequately from those of another construct. Our results opposed to some of the research that has suggested (Božinović and Sindik, 2017) that there is no clear line between strategy dimensions. In our results strong correlations were found between the factors; this stems from the fact that our scale measures the same construct, namely LLS. The highest correlation was reported between the sociocultural-interactive and meta-sociocultural-interactive fields, while the correlation between the sociocultural-interactive and cognitive fields fell lower – though it was also noteworthy. At the same time all fields were distinct from each other that demonstrate the strong interdependence of the different factors and the fact that the strategies are distinct in a sense yet still interrelated and interwoven.

Due to the fact that the sample showed moderate use of each of the strategies, we suppose that the students use a certain set of strategies drawn from the various fields. Their choice depended on their personality, their age and the educational traditions in their country. For example, the reported use of meta-affective strategies turned out to be significantly different in the older age group, with Year 6 showing significantly lower use of the affective factors. We should also state that the research concentrated on the differences between the years. It was not our aim to examine gender differences. Other research has also concluded that strategy preferences are personally and culturally dependent and can vary greatly (Wang et al., 2013; Božinović and Sindik, 2017; Oxford, 2017; Chen and Lin, 2018). For this particular age group, the affective field had to be merged with other factors, implying that the number and content of the statements in this dimension were not sufficient to characterise the construct of the affective aspect. However, the affective factor is a very important field for this age group; therefore, the scale should be revised and reconstructed using more differentiated and diverse statements that can describe the affective construct more thoroughly.

In conclusion, our study validated a scale that represents self-regulated language learning strategy use in lower secondary school students in Hungarian context. However, when using this scale for a different group, it would be necessary to conduct another investigation of the metric characteristics of the instrument. These investigations can result in a somewhat diverse structure and strategy classification, which is characteristic of that particular sample. The factor structure can be modified depending on the various samples.

It follows from these facts that our study also has certain limitations. First, we only identified five strategy fields and were not able to include the affective factors. We know from previous research that the effect of affective factors is considerable. Our next goal is to review how we can involve the affective aspect of language learning.

Second, self-report questionnaires can be used to evaluate and monitor one’s own learning activity in different ways. In addition, the fields measured are so closely related that it is difficult to ascertain the boundaries. We see so much after this research that we believe that we would now be able to conduct an even more precise evaluation of the fields.

Third, our research could not eliminate the effect of other foreign languages. While we only analysed English as a foreign language, the children may have been learning other languages as well in or out of school. It can thus be assumed that they may not have been able to match the different foreign languages they were learning with the various language learning strategies they were using for each.

Fourth, we only involved lower secondary school children in Hungary. Hence, our results can only be generalised to other countries through further research. In addition, expanding the use of the questionnaire to other age groups could confirm whether the questionnaire is a reliable tool for other populations as well.

Finally, the aim of this research was to develop a scale for self-regulated language learning strategy use; individual differences, such as gender, age, socio-economic status and other variables were therefore not taken into consideration. Our intention is to include these factors in future research to provide more evidence for the validity of the questionnaire.

## Conclusion

On the whole, we have found that our questionnaire comprises important constructs to measure the use of self-regulated foreign language learning strategies in the observed sample. The main significance of our research is that it provides empirical evidence for the viable transfer of SRL theory from educational psychology to EFL teaching and offers evidence that it is possible to design a self-reported scale that can be used to measure lower secondary children’s self-regulated language learning.

The main advantage of our research is that our questionnaire can be employed in classroom environments and that it provides immediate feedback to both students and teachers on various aspects of students’ language learning processes. The research also explores ways to discover further aspects of language learning processes and highlights pathways for students on how to become self-directed and more efficient language learners.

## Pedagogical Implications

As for the pedagogical implications, our results highlight the importance of SRL research in foreign language teaching and confirm the importance of implementing language learning strategies in foreign language instruction. There are also some effective efforts to develop students’ self-regulated strategies with strategy trainings embedded in education courses, thus confirming the significance of strategy use in learning (De la Fuente et al., 2015; Minnaert et al., 2017; Oxford and Amerstorfer, 2018). The study shows teachers that appropriate strategy use does not only mean the ability to choose from among several techniques and methods, but also contains a self-regulated feature that is manifested in the children regulating their own learning processes and thus taking responsibility for their own development. Practically, the Self-Regulated Foreign Language Learning Strategy Questionnaire may be useful for lower secondary teachers of foreign languages as a self-evaluation tool to assess their students’ level of consciousness of their own self-regulated learning and to raise their students’ awareness of strategy use in language learning. Although it is only concerned with five aspects of self-regulated learning strategies, it can provide a comprehensive view of students’ preferences and can serve as a basis for future strategy instruction.

## Ethics Statement

This study was carried out in accordance with the recommendations of the University of Szeged. Participating schools obtained written informed consent from the children’s parents. The research strictly adhered to the usual standards of research ethics as approved by the IRB of the Doctoral School, University of Szeged.

## Author Contributions

AH and AM contributed equally to the research design, implementation and reporting of this research. AH and AM wrote and reviewed the manuscript, and approved the final version.

## Conflict of Interest Statement

The authors declare that the research was conducted in the absence of any commercial or financial relationships that could be construed as a potential conflict of interest.

## Appendix

**Table:** Self-Regulated Foreign Language Learning Strategy Questionnaire (Srfllsq).

When I learn English, …
**Metacognitive**
I think of the relationships between what I already know and new things I learn in English.
I first skim an English passage, then go back and read carefully.
I look for opportunities to read as much as possible in English.
I write notes, messages, letters, or reports in English.
I plan my schedule so I will have enough time to study English.
I pay attention when someone is speaking English.
I make summaries of information that I hear or read in English.
I try to find out how to be a better learner of English.
**Cognitive**
I connect the sound of a new English word and an image or picture of the word to help me remember the word.
I use the English words I know in different ways.
I find the meaning of an English word by dividing it into parts that I understand.
I use new English words in a sentence so I can remember them.
I try to find patterns (grammar) in English.
I try not to translate word for word.
**Meta-affective**
I notice if I am tense or nervous when I am studying or using English.
I encourage myself as I learn English so that I can learn what I would like.
I read in English as a leisure-time activity.
I organise my English language learning so that I always enjoy doing it.
I plan my English language learning so that I can perform better.
I have more success learning English when I feel like doing it.
I give myself a reward or treat when I do well in English.
I try to relax whenever I feel afraid of using English.
**Meta-sociocultural-interactive**
I try to learn about English-language cultures and/or other cultures through English.
I look for people I can talk to in English.
I look at English-language TV shows, movies or websites to get to know the cultures of English native speakers and/or other cultures through English.
I choose leisure activities where I encounter English-language cultures and/or other cultures through English as well.
I plan what I want to find out about the cultures of English speakers and/or other cultures through English.
I practise English with my peers.
I look for similarities and differences between my own culture and the cultures of English native speakers and/or other cultures through English.
Getting to know English-language cultures helps me to learn the language.
**Sociocultural-interactive**
I start conversations in English.
I make up new words in English if I do not know the right ones.
When I speak with highly proficient speakers of English, I think it is important to get acquainted with their culture.
I encourage myself to speak English even when I feel afraid of making a mistake.
